# Rifampicin Resistance in Tuberculosis Outbreak, London, England

**DOI:** 10.3201/eid1106.041262

**Published:** 2005-06

**Authors:** Claire Jenkins, Alleyna P. Claxton, Robert J. Shorten, Timothy D. McHugh, Stephen H. Gillespie

**Affiliations:** * Royal Free Hospital, London, United Kingdom;; † Homerton Hospital, London, United Kingdom

**Keywords:** multi-drug resistant Mycobacterium tuberculosis, sequencing, rare mutations

## Abstract

*Mycobacterium tuberculosis* isolates cultured from 6 patients associated with an isoniazid-resistant *M. tuberculosis* outbreak acquired rifampicin resistance. The *rpoB* gene sequence showed that resistance was associated with rare mutations in each isolate. Three isolates had a mutation outside the rifampicin resistance–determining region.

In January 2000, 4 cases of smear-positive pulmonary tuberculosis (TB) caused by *Mycobacterium tuberculosis* in young men from the local community were identified during a 1-week period at a hospital in north London, United Kingdom (1). Three isolates were shown to be isoniazid-monoresistant TB. Further investigation showed 155 confirmed or probable cases, 132 in London and 23 outside London, which suggests a large outbreak of a unique strain in the London area (2). Confirmed case-patients were defined as patients with isolates of *M. tuberculosis* resistant to isoniazid that had the same band pattern on restriction length polymorphism typing (RFLP); these patients were residents of London at the time of their diagnosis, which had been made since January 1995 (2). Probable cases were defined as for confirmed cases except that isolates underwent rapid epidemiologic typing [RAPET] but are awaiting RFLP typing (2). RAPET is a rapid screening molecular typing method developed at the Mycobacterium Reference Unit (MRU) (3). In October 2002, the TB isolate from 1 patient associated with the outbreak had developed resistance to rifampicin. Initially, the isolate was tested with a commercial line probe rifampicin resistance–determining hybridization assay, Inno-LiPA (Innogenetics Belgium, Gent, Belgium). However, the line probe assay failed to identify rifampicin resistance in this isolate, and rifampicin resistance was only detected on phenotypic antimicrobial sensitivity testing at MRU. Subsequently, 5 additional rifampicin-resistant isolates from different patients associated with this outbreak were identified at MRU. The aim of this study was to determine the basis for the rifampicin resistance in these 6 isolates by sequencing the entire *rpoB* gene.

Rifampicin resistance can occur as a result of mutations on the *rpoB* gene that encodes the β-subunit of RNA polymerase (4). More than 95% of these mutations occur on an 81-bp fragment of the gene between bases 1276 and 1356 (432–458 in the *rpoB* gene of *M. tuberculosis* and codon 507–534 in the *Escherichia coli rpoB* gene) (5,6). This region is known as the rifampicin resistance–determining region (RRDR), or "hotspot," and is used as a target for direct sequencing and commercial line probe assays.

## The Study

The isoniazid-monoresistant and multidrug-resistant tuberculosis (MDR-TB) isolates were obtained from the patients' source hospital or MRU. All isolates were identified as belonging to the same strain of *M. tuberculosis* (RAPET or IS*6110* typing), and all drug-susceptibility testing was carried out at MRU according to standard procedures. The wildtype control isolate used for these studies was *M. tuberculosis*, H37Rv (ATCC, 9360 National Collection of Type Culture, London, UK). Three isolates, 018, 483, and 915, were from patients in whom MDR-TB developed as a result of poor compliance with therapy, whereas isolates 604, T7, and 371 were from patients who contracted primary MDR-TB.

DNA was prepared from the 6 isolates of *M. tuberculosis* by emulsifying 2–3 colonies in 400 μL Tris-EDTA buffer and heating the suspension in a water bath for 40 min at 80°C. Polymerase chain reaction (PCR) was performed on the extracted DNA with 6 sets of primers designed to amplify 6 overlapping fragments of the *rpoB* gene from the 6 *M. tuberculosis* isolates (Table). Ten microliters of DNA was added to the PCR mix containing 81.4 μL PCR-quality water, 10 μL potassium chloride buffer (Bioline Ltd., London, UK), 0.4 μL of each primer (100 mmol) (Sigma-Genosys Ltd., Haverhill, UK), 3 μL deoxynucleoside triphosphates (5 mmol), and 1 μL Taq (Bioline). The amplification was performed on a Techgene thermal cycler (Techne, Princeton, NJ, USA). PCR products were separated by gel electrophoresis on a 1.5% agarose gel, and DNA bands were stained with ethidium bromide. Primers and excess nucleotides were removed from the amplified DNA with a PCR clean-up kit (Qiagen, Inc., Valencia, CA, USA). The amount of DNA in the cleaned-up product was quantified by comparing the intensity of the band to bands of known intensity in a HyperLadder marker (Bioline).

**Table:** Sequences of 6 sets of primers designed to amplify 6 overlapping fragments of the *rpoB* gene from *Mycobacterium tuberculosis* isolates

Fragment	Amplicon size	Primer position	Primers
1	697 bp	–135 to –112* 541–562	Forward 5´ GTT TAG TTG CGT GCG TGC Reverse 5´ CTTGTCAAT GGT CTC GTC GAA
2	688 bp	451–472 1119–1139	Forward 5´ TTC CCG ATG ATG ACC GAG AAG Reverse 5´ GGA TCA GCT CGC CGA CCG TA
3	706 bp	1029–1048 1714–1735	Forward 5´ CGA GGG TCA GAC CAC GAT G Reverse 5´ GCG GGG CGA GAC GTC CAT GTA
4	681 bp	1624–1645 2284–2305	Forward 5´ ATC GAT GCG GAC GGT CGC TTC Reverse 5´ CGG GAT GTC GCG GGT GAT CTC
5	811 bp	2194–2215 2887–3005	Forward 5´ TCC AAC CGC CTG GTC GAA GAG Reverse 5´ CGT CGA CAC AAT GGC GTT
6	847 bp	2797–2815 +113 to +135*	Forward 5´ TGT GCC CAC AGC GGC TGG Reverse 5´ CTT TTT GAC CTC GCC ATA GGA C

*Denotes a primer position in the sequence before the start (–) or after the end (+) of the *rpoB* gene sequence.

Forward and reverse cycle sequencing reactions were performed with the Big Dye Terminator Cycle Sequencing Ready Reaction DNA sequencing kit (Applied Biosystems, Inc., Foster City, CA, USA). Briefly, 40 ng of cleaned-up DNA was added to 10.8 μL PCR-quality water, 3 μL buffer, 3.2 μL of forward or reverse 1 mmol primer, and 1 μL of cycle sequencing ready reaction mix. The labeled DNA was precipitated by adding 14.5 μL PCR-quality water, 62.5 μL 95% ethanol, and 3 μL sodium acetate solution (2.3 mol/L) and centrifuging (13,000 × *g*, 15 min, 4°C). The supernatant was removed with a fine-tipped pipette, and the pellet was cleaned with 200 μL 70% ethanol and then recentrifuged (13,000 × *g*, 15 min, 4°C). Again the supernatant was removed, and the pellet was dried at 37°C for 30 min. Four microliters of formamide and 1 μL of dextran loading buffer were added to each pellet, and 1.5 μL of sample was added to each well of the sequencing gel. The 6 fragments of the *rpoB* gene from each of the 6 isolates were then sequenced with an ABI 377 Applied Biosystems sequencer. The sequences obtained from each isolate were joined together to form a continuous whole gene sequence, aligned with ClustalW (http://www.ebi.ac.uk/clustalw/) and compared to the wildtype to identify base-pair mismatches.

In addition, all 6 isolates were tested with a line probe resistance–determining hybridization assay, Inno-LiPA (Innogenetics Belgium), as described in the manufacturer's instructions. Briefly, an 81-bp region of the *rpoB* gene was amplified with biotinylated primers, which yielded a biotinylated target sequence, and hybridized with specific oligonucleotide probes immobilized on a parallel strip. After hybridization, streptavidin labeled with alkaline phosphatase was used to detect any hybrids. Inno-LiPA consists of 10 oligonucleotide probes (19–23 bases in length), encompassing the 81-bp region (RRDR) of the *rpoB* gene. One is specific for *M. tuberculosis* complex, whereas the other 5 partially overlapping wildtype probes (S1–S5) cover the region from positions 507 to 534 of the *rpoB* gene. These S-probes hybridize to the wildtype (rifampicin-sensitive) DNA sequence. Failure of any of these S-probes to hybridize indicates that a mutation has occurred. Four other probes (R2, R4a, R4b, and R5) are specific for amplicons carrying the most common *rpoB* mutation that confers rifampicin resistance.

The results of the Inno-LiPA assay showed 5 of the 6 isolates, which were phenotypically rifampicin-resistant, were negative. The target DNA hybridized to all 5 wildtype S-probes and none of the R-probes, which demonstrated that the assay failed to detect rifampicin resistance in these 5 isolates. The Inno-LiPA assay cannot detect mutations outside the RRDR. Failure to detect rare mutations within the RRDR may be caused by nonspecific hybridization of the wildtype S-probes because of slight fluctuations in temperature during the hybridization process. Isolate 483 showed a weak DNA hybridization reaction with the S4 probe, indicating that a mutation was present on codon 451 (codon 526 in *Escherichia coli*). The exact nature of the mutation could not be determined with this assay.

**Figure 1:**
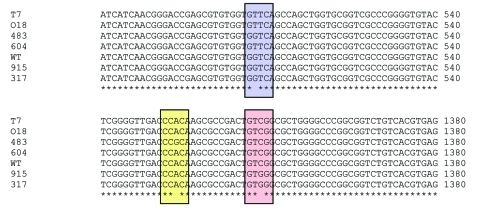
Alignment of *rpoB* gene sequences showing the mutations detected in this case study. WT, wildtype; T7, 018, 604 – V176F (G to T, highlighted in blue); 483, H526R (A to G, highlighted in yellow); 915, 317, S531W (C to G, highlighted in pink).

Analysis of the sequence data identified specific mutations in *rpoB* in all 6 strains studied (Figure 1). Three had mutations within the RRDR, and of these, 2 were C-to-G mutations at codon 456, inducing a serine to tryptophan amino acid conversion (S456W). The third was an A-to-G mutation at codon 451, resulting in a change from histidine to arginine (H451R) (Figure 1). No other mutations were identified in the DNA sequences outside the RRDR in these 3 isolates. The 3 other isolates had the mutation G to T at codon 176, outside the RRDR, which caused a change from valine to phenylalanine (V176F). Neither RRDR mutations nor any other mutations were found in the *rpoB* gene sequences of these 3 isolates.

Of the mutations found within the RRDR, H526R occurs in <4%, and S531W occurs in ≈1.4% of all rifampicin-resistant isolates, respectively (7). Mutations outside the RRDR account for <4% of rifampicin resistance, and few have been described (7–9). In a previous study (7), V176F was found in 5 of 18 isolates with no mutations in the RRDR from Asia (9,10) and Africa (11,12). To our knowledge, this is the first time this type of mutation has been detected in strains isolated in the United Kingdom.

**Figure 2:**
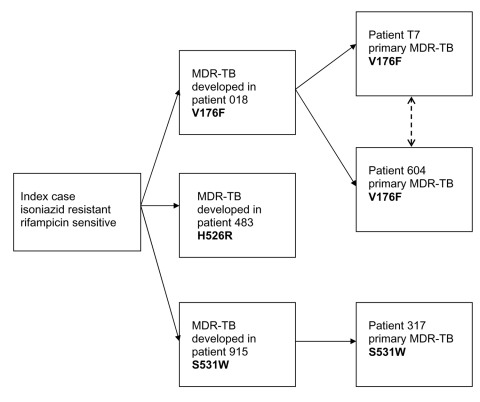
Suggested epidemiologic relationship between 6 cases of multidrug-resistant tuberculosis (MDR-TB). Resistance to rifampicin developed in patients 018, 483, and 915 while on therapy, whereas patients T7, 604, and 317 contracted primary MDR-TB. The type of mutation present in each patient's strain of MDR-TB is highlighted in bold.

Three different mutations in the *rpoB* gene, V176F, H526R and S531W, were detected in the isolates associated with the isoniazid-resistant outbreak. Given that all the mutations found in this study are rare and that MDR-TB developed during treatment in 3 patients (018, 483, and 915) (Figure 2), the rifampicin-resistance mutations observed in these 3 patients likely occurred on 3 independent occasions, as a result of poor compliance with therapy. Two of these patients, 018 and 915, were contacts of the 3 patients who subsequently had primary MDR-TB (patients T7 and 604 [V176F] and 317 [S531W]) (Figure 2).

## Conclusions

Our study highlights problems associated with using a line probe hybridization assay to detect rifampicin resistance in *M. tuberculosis*. The Inno-LiPA assay cannot detect mutations outside the RRDR, and failure to detect rare mutations within the RRDR may be caused by nonspecific hybridization of the wildtype S-probes because of slight fluctuations in temperature during the hybridization process. In this study, all the rare mutations, inside and outside the RRDR, were detected by sequencing the entire *rpoB* gene. The inability to identify MDR-TB isolates results in treatment failure and increased risk for transmission of resistant disease in the community. We recommend that, when the index of suspicion for MDR-TB is high and the line probe assays fail to detect mutations conferring rifampicin resistance, the entire *rpoB* gene should be sequenced to prevent unnecessary delay in diagnosing MDR-TB.
